# Cuticular Membrane of *Fuyu* Persimmon Fruit Is Strengthened by Triterpenoid Nano-Fillers

**DOI:** 10.1371/journal.pone.0075275

**Published:** 2013-09-24

**Authors:** Shuntaro Tsubaki, Kazuki Sugimura, Yoshikuni Teramoto, Keizo Yonemori, Jun-ichi Azuma

**Affiliations:** 1 Science Research Center, Kochi University, Kochi-shi, Kochi, Japan; 2 Graduate School of Agriculture, Kyoto University, Kitashirakawa Oiwake-cho, Sakyo-ku, Kyoto, Japan; 3 Faculty of Applied Biological Sciences, Gifu University, Gifu, Japan; 4 Graduate School of Engineering, Osaka University, Suita, Osaka, Japan; Dalhousie University, Canada

## Abstract

The mechanical defensive performance of fruit cuticular membranes (CMs) is largely dependent on the molecular arrangement of their constituents. Here, we elucidated nano-sized interactions between cutin and triterpenoids in the cuticular matrix of *Fuyu* persimmon fruits ( *Diospyros* *kaki* Thunb. cv. *Fuyu*), focusing on the mechanical properties using a combination of polymer analyses. The fruit CMs of *Fuyu* were primarily composed of wax (34.7%), which was predominantly triterpenoids followed by higher aliphatic compounds, and cutin (48.4%), primarily consisting of 9,10-epoxy-18-hydroxyoctadecanoic acid and 9,10,18-trihydroxyoctadecanoic acid. Based on the tensile tests of the CM, the removal of wax lead to a considerable decrease in the maximum stress and elastic modulus accompanied by an increase in the maximum strain, indicating that wax is of significant importance for maintaining the mechanical strength of the CM. Wide-angle X-ray diffraction and relaxation time measurements using solid-state ^13^C nuclear magnetic resonance indicated that the triterpenoids in the cuticular matrix construct a nanocomposite at a mixing scale below 20-24 nm; however, the higher aliphatic compounds did not exhibit clear interactions with cutin. The results indicated that the triterpenoids in the cuticular matrix endow toughness to the CM by functioning as a nanofiller.

## Introduction

The plant cuticular membrane (CM) is a vital protective barrier and structural support for the surface of green plants, allowing them to withstand various types of mechanical stress [1]. The mechanical durability of their CMs is also important for the quality of fruits, impacting their surface appearance and shelf life [2]. CMs are complex materials composed of cutin, waxes, polysaccharides and cutan [3]. Cutin provides the structural matrix of the CM and consists of polyesters of hydroxylated and epoxy-hydroxylated fatty acids with chain lengths of C16 and C18. Waxes are a mixture of lipophilic compounds embedded inside the cutin network and are also deposited on the exterior of the cutin matrix, providing water repellency. The constituents of waxes include a wide variety of fatty acids, primary alcohols, aldehydes, *n*-alkanes, esters and pentacyclic triterpenoids [4]. CMs also contain cell-wall polysaccharides, such as cellulose, hemicelluloses and pectin [5], which exert a rigidizing effect [6]. Some plant CMs include a highly recalcitrant material called cutan that forms networks with polymethylenic chains and ether linkages, providing high tolerance against biodegradation [7].

The CMs of tomato fruits have been extensively studied as a model material for characterizing their physical toughness [8]. Mechanical analyses, such as tensile tests and load-creep tests, have been conducted to evaluate the mechanical properties of CMs [2,9]. The accumulation of polysaccharides, waxes and phenolics in tomato CMs together with their development are associated with improvements in their elasticity and stiffness [1,10-13]. The monomeric composition and the numbers and types of cross-linkages of cutin also affect the mechanical behavior of CMs [1]. Environmental conditions (e.g., humidity and temperature) dynamically modify the extensibility and plasticity of CMs [14]. Moreover, different plant species and organs exhibit different mechanical behaviors; for instance, the CMs of *Yucca* *aloifolia* L., *Hedera* *helix* L., *Nerium* *oleander* L. and *Sonneratia* *alba* J. Smith leaves have a wide distribution of elastic modulus, maximum stress and maximum strain values that are specific to each plant, with considerable differences from the values reported for tomato CMs [6,15,16].

The presence of great diversity in species, organ-dependent morphologies and compositions of plant CMs motivates research for obtaining further insight into the structural arrangement of the constituents in CMs from different plants. Among the plant stuffs investigated to date, persimmon fruits have greater advantages for providing uniform, thick and flexible CMs for testing of their mechanical properties, including tensile tests and dynamic mechanical analyses. We recently found that an increase in the CM density has a significant correlation with increases in the elasticity and strength, which are accompanied by a decrease in viscoelasticity, of CMs by analyzing 27 cultivars of persimmon fruits [17]. The CM density has been hypothesized to be associated with an accumulation of lower molecular weight components, such as waxes and phenolics, in the cutin network, and these components reduce the segmental mobility of the cutin matrix and increase the mechanical strength of the CMs in a manner similar to fillers [1,13]. The arrangement of wax compounds has been hypothesized to influence the cuticular functions [18]; however, the role of cuticular waxes and their compositions on the biomechanics of CMs have not been thoroughly analyzed [1]. The high wax content (21.7-38.0% w/w) and low polysaccharide content (0.6-11.2% w/w) in persimmon fruits warrants an investigation on the contribution of wax to the mechanical properties of fruit CMs [17].

In this study, we evaluated the nano-composite structure of cutin and triterpenoids of the *Fuyu* persimmon fruit CM ( *Diospyros* *kaki* L. Thunb. cv. *Fuyu*), which is one of the most widely grown commercial non-astringent persimmons.

## Materials and Methods

### Materials

Mature *Fuyu* persimmon fruits ( *Diospyros* *kaki* Thunb. cv. *Fuyu*) were obtained from the Experimental Fruit Garden, Graduate School of Agriculture, Kyoto University, in November of 2011 and 2012.

A mixture of authentic standards of *n*-alkanes (C21-C40) and standard compounds for GC/MS analysis (hexacosanoic acid, octacosanoic acid, hexacosanol, octacosanol, triacontanol, β-sitosterol, oleanolic acid, and ursolic acid) were purchased from Sigma-Aldrich, St. Louis, MO, USA and the Tokyo Chemical Industry Co., Ltd, Tokyo, Japan, respectively.

### Isolation of cuticular membranes

Peels were manually striped and immersed in a mixture of pectinase (2.0% w/w, *Aspergillus niger*, Sigma-Aldrich, St. Louis, MO, USA) and meicelase (0.5 to 1.0% w/w, Meiji Seika Kaisha Ltd., Tokyo, Japan) for more than 2 days at 37 °C to isolate the CMs. The isolated CMs were washed with distilled water and dried at 45 °C for 2 days. The surface area of the CMs was measured using Photoshop CS5 (Adobe Systems Inc., CA, USA). The thicknesses of the CMs were measured using a digital micrometer (DP-1 VR, Mitutoyo Co., Tokyo, Japan), and the mean value of three measurements per specimen was recorded as the thickness. The density (mg/cm^3^) of the CMs was determined by dividing the CM weight per unit area (cm^2^) by the thickness.

### Gravimetric analysis of general CM composition

**Figure 1:**
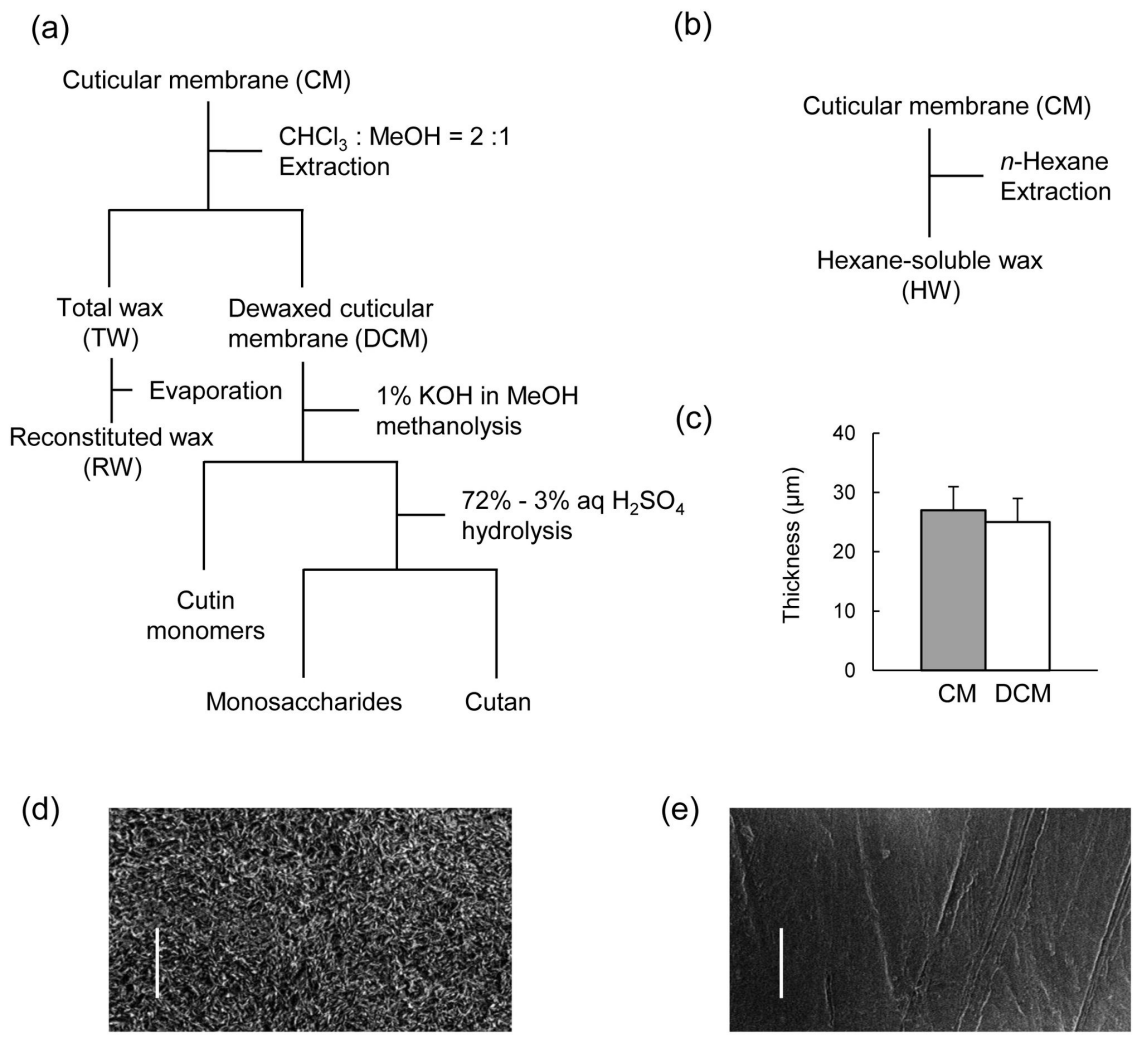
Preparation of cuticular membrane (CM) from *Fuyu* persimmon fruits. (a) Schematic diagram for the preparation of total wax (TW), reconstituted wax (RW), cutin, monosaccharide and cutan. (b) Schematic diagram for the preparation of hexane-soluble wax (HW). (c) Thicknesses of the CM and dewaxed cuticular membrane (DCM) determined using a micrometer. (d) Scanning electron microscopy (SEM) image of native CM. (e) SEM image of DCM. Bars indicate 10 µm.

The chemical composition of the CMs was gravimetrically determined according to the procedures of Domínguez et al. [13], Takahashi et al. [6] and Tsubaki et al. [17]. Four specimens obtained from different *Fuyu* fruits were used for the analyses. The sequential extraction procedure is summarized in Figure 1a and b. The total wax (TW) contents were determined by measuring the differences in weight after triplicate extractions with a mixture of chloroform/methanol (2/1, v/v) at 50 °C for 2 h. The degree of removal of TW was confirmed by low-voltage scanning electron microscopy (LV-SEM, VE-8800, Keyence Co.). The content of non-polar wax was determined from the weight changes after triplicate extractions using *n*-hexane at 50 °C for 30 min (hexane-soluble wax; HW). The isolated TW was further evaporated to dryness using an evaporator at 40 °C to produce reconstituted wax (RW) for chemical and physical analyses. The cutin content was determined from the weight changes following methanolysis of the dewaxed cuticular membrane (DCM) produced after the wax extraction of CM with 1% potassium hydroxide in methanol at 80 °C for 2 h in triplicate. The polysaccharide contents in the residue following methanolysis were determined using the phenol-sulfuric acid method after a 2-step acid hydrolysis; namely, the sample was mixed in 72% sulfuric acid and maintained at room temperature for 1 h, followed by diluting with distilled water to 3% H_2_SO_4_ and autoclaving at 120 °C for 1 h. The mixed standard solution of arabinose, xylose, glucose, galactose and mannose in a ratio determined by high-performance anion exchange chromatography (HPAEC, described in a later section) were used to generate the calibration curve. The final residues following acid-hydrolysis were defined as cutan.

### Cutin analysis

The monomeric composition of cutin in the fruit CM was determined following depolymerization via hydrogenolysis according to the method of Walton et al. [19] using LiAlH_4_ in THF. Reduction was also performed using LiAlD_4_ to label the carboxyl and epoxy groups in the cutin monomers with deuterium. Excess reagents were degraded by the addition of distilled water. The solution was then acidified with HCl, and the hydrogenolysate and deuteriolysate were extracted with diethyl ether. The extracts were dried over anhydrous sodium sulfate and evaporated to dryness. The trimethylsilyl ethers of the cutin monomers were obtained by treatment with BSA (N,O-bis(trimethylsilyl)-acetamide) and analyzed by GC/MS (Shimadzu GC-2010/PARVUM2 system, EI; 70 eV, Shimadzu Co., Kyoto, Japan) equipped with a DB-1 capillary column (J&W Scientific, 0.25 mm × 30 m, df = 0.25 µm, Agilent Technologies, Inc., Santa Clara, CA). The oven temperature was programmed as follows: 195 °C to 240 °C at 2 °C/min, maintained at 240 °C for 10 min and then heated to 300 °C at 10 °C min^-1^. Cutin monomers were identified by comparing the mass spectrum with published spectra [19]. The compositions of the cutin monomers were expressed as a relative peak area (%) compared to the total peak area that appeared in the total ion chromatogram.

### Wax analysis

Wax (TW and HW) was extracted following the above-mentioned procedure. The extracted waxes were trimethylsilylated using BSTFA (N,O-Bis(trimethylsilyl) trifluoroacetamide, Tokyo Chemical Industry Co., Ltd., Tokyo, Japan), and the trimethylsilylated derivatives were analyzed by gas chromatography-mass spectrometry (GC/MS) using a DB-5 column (J&W Scientific, 0.25 mm × 30 m, df = 0.25 µm, Agilent Technologies, Inc., Santa Clara, CA, USA) with the following oven temperature program: 180 °C to 320 °C at 3 °C min^-1^ (20 min) [20-22]. The components were identified using a combination of NIST 05 MS spectral data and authentic standards of alkanes, alcohols, fatty acids, and triterpenoids, as shown in the materials section. The composition of the wax was expressed using the same method as for cutin monomers.

### Monosaccharide analysis

The monosaccharide compositions obtained after Saeman hydrolysis of the decutinized DCMs were determined by high performance anion exchange chromatography (HPAEC, DX-500, Dionex, Sunnyvale, CA, USA) equipped with a pulsed amperometric detector and a CarboPac PA-1 column (column size: 4.0 × 250 mm, Dionex), using 1.0 mM aqueous NaOH as an eluent at a flow rate of 1.0 mL min^-1^, as previously reported [23].

### Nuclear magnetic resonance (NMR) analyses

The CMs were analyzed by solid state ^13^C nuclear magnetic resonance (NMR) spectroscopy using a Varian 400 MHz NMR system (100.55 MHz, Agilent Technologies, Inc., Santa Clara, CA, USA). Cross-polarization magic angle spinning (CP/MAS) ^13^C-NMR spectra were obtained to characterize the carbons in the CM, DCM and RW by 2 ms of contact time and 2.9 µs of a 90° pulse width. Proton spin-lattice relaxation times in the rotating frames (*T*_1ρ_^H^) were measured using 0.05-10 ms of spin-locking time with 0.2 ms of contact time. Proton spin-lattice relaxation times in the laboratory frame (*T*_1_^H^) were measured using a π-τ-π/2 pulse sequence with different durations of delay times, from 0.1 to 3 s, with 2 ms of contact time. All the NMR experiments were conducted using a MAS rate of 15 kHz at 20 °C with at least 2048 scans, and the chemical shifts were referenced to adamantine.

The *T*_1ρ_^H^ values of each carbon in CM, DCM and RW were determined by fitting the carbon resonance intensities to the following equation between the range of spin-lock times from 0.05 to 10.0 ms:

$$\frac{M(\tau )}{M(0)}=x_{a}\exp\left(\frac{-\tau }{T_{1\rho }^{Η},fast}\right)+x_{c}\exp\left(\frac{-\tau }{T_{1\rho }^{Η},slow}\right)$$ (1)

where *M*(*τ*), *M*(0), τ, *T*_1ρ_^H^_fast_ and *T*_1ρ_^H^_slow,_ *x*_a_ and *x*_c_ indicate the normalized magnetization intensity, maximum magnetization intensity, spin-locking time, relaxation times in the mobile and rigid components and the weight fractions of the domains, respectively. The *T*_1_^H^ values were determined by fitting the signal intensity to the following equation using different delay times from 0.1 to 3.0 s:

$$M(\tau )=M_{\infty }\left[1-2\exp\left(\frac{-\tau }{T_{1}^{Η}}\right)\right]$$ (2)

where *M*_∞_ is the intensity of the resonance signal at τ ≥ 5*T*_1_^H^. The effective domain sizes (*L*) of cutin and wax were estimated using the following equation:

$$L\approx {\left[6DT_{i}^{Η}\right]}^{\frac{1}{2}}$$ (3)

where *D* and *T*_i_^H^ indicate the spin-diffusion coefficient (a value of 1 × 10^-16^ m^2^ s^-1^ was used in this study) and *T*_1ρ_^H^ and *T*_1_^H^, respectively.

### Wide-angle X-ray diffraction analysis

Wide-angle X-ray diffraction (WAXD) measurements were performed over a temperature range of −100 to 300 °C using a Rigaku Ultima-IV diffractometer (Rigaku Co., Tokyo, Japan) equipped with an attachment for medium- and low-temperature diffractometry. Nickel-filtered CuKα radiation (λ = 0.1542 nm) was used at 40 kV and 40 mA. The temperature was controlled using a Rigaku PTC-30 temperature controller. After reaching the given temperature by sweeping the temperature at 3 °C min^-1^, the temperatures of the specimen were stabilized for 5 min. Then, the diffraction intensity profiles were collected in the range of 2θ = 2 to 40° with a 0.02° min^-1^ step. The minimum sizes of the wax crystals (*D*) were estimated using Scherrer’s equation (4).

$$D=\frac{0.9\times \lambda }{\beta \cos\theta }$$ (4)

where λ, β and *θ* are the X-ray wavelength, line broadening at half of the maximum intensity and the Bragg angle, respectively.

### Mechanical tests

Tensile tests were conducted using a rheometer (Rheogel E4000, UBM, Kyoto, Japan). The CMs were cut into rectangular shapes (0.5 × 2.0 cm) and attached to the clamps of the rheometer at an extension speed of 55 µm s^-1^ at 23 °C until failure. The maximum values of strain and stress before failure of the CM sheet are reported as the maximum strain (%) and the maximum stress (MPa). The elastic modulus (MPa) was obtained from the slope of the linear region of the stress-strain curve with the least-squares method. Faultless segments (*n* = 5-9) cut from 3 fruits were measured, and the difference before and after wax extraction was evaluated using a one-sided *t*-test.

The temperature dependency of the storage elastic modulus (*E*′) and loss elastic modulus (*E*′′) were analyzed using a dynamic mechanical analyzer (DMA) (Seiko DMS6100/EXSTAR6000) over the temperature range from -60 °C to 95 °C with a scanning rate of 2 °C min^-1^ and an oscillatory frequency of 10 Hz with a 0.5 × 1.5 cm span length. Decreases in the storage elastic modulus (*E*’) and loss elastic modulus (*E*”) indicate elasticity and viscoelasticity, respectively.

## Results

### Chemical constituents of *Fuyu* persimmon *Fuyu* CM

**Figure 2:**
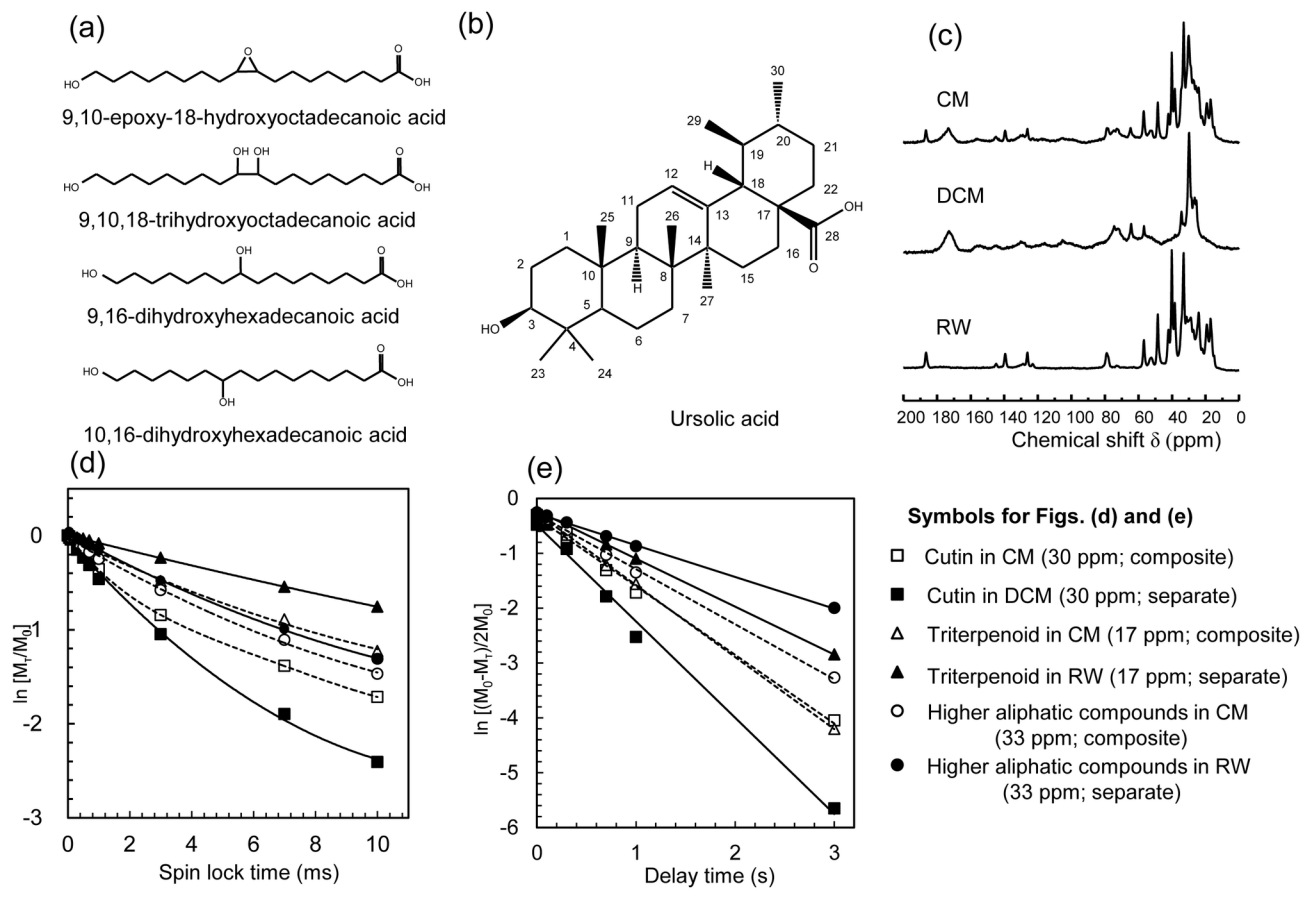
NMR analyses of cuticular membrane (CM) of *Fuyu* persimmon fruits. (a) Chemical structures of cutin monomers and (b) ursolic acid. (c) Solid state CP/MAS ^13^C-NMR spectra of the CM, dewaxed CM (DCM) and reconstituted wax (RW) obtained at a MAS rate of 15 kHz. (d) Logarithmic plots of the intensities of signals at 17 ppm (triterpenoids in wax), 30 ppm (cutin) and 33 ppm (higher aliphatic compounds in wax) of CM, DCM and RW for measuring the proton spin-lattice relaxation time in the rotating frame (*T*_1ρ_^H^). (e) Logarithmic plots for the proton spin-lattice relaxation time in the laboratory frame (*T*_1_^H^) (selected signals are the same as that for *T*_1ρ_^H^).

The isolated fruit CM of *Fuyu* weighed 1.66 mg cm^-2^, and its most predominant constituent was cutin, amounting to 48.4% (w/w) (Table 1). The major monomeric constituents of the cutin were 9,10-epoxy-18-hydroxyoctadecanoic acid (43.7% w/w) and 9,10,18-trihydroxyoctadecanoic acid (31.3% w/w) (Table 2 and Figure 2a). These results indicated that the C18 monomers were the major building units of the CM framework.

**Table 1:** Chemical composition of CMs isolated from *Fuyu* persimmon fruits.

Components	Weight per unit area (μg cm^-2^)
Total wax (TW)	576 ± 28 (34.7%)
*n*-Hexane-soluble wax (HW)	183 ± 9 (11.0%)
Cutin	803 ± 35 (48.4%)
Polysaccharide	45 ± 6 (2.7%)
Cutan	34 ± 8 (2.0%)
Total	1660

Values are expressed as the mean value ± SD (*n* = 4). Values in parentheses indicate the relative composition on a weight basis.

**Table 2:** Composition of cutin isolated from the CM of *Fuyu* persimmon fruits.

*Rt* (min)	Component	Corresponding cutin monomer	Composition (Relative area %)
19.3	Hexadecane-1,7 (8),16-triol	9 (10),16-dihydroxyhexadecanoic acid	17.4 ± 0.7
23.5	Octadecenetriol ^a^	9,10-epoxy-18-hydroxyoctadecanoic acid^a^	1.0 ± 0.1
24.3	Octadecenetriol ^a^	9,10-epoxy-18-hydroxyoctadecanoic acid^a^	2.7 ± 0.1
24.9	Octadecenetriol ^a^	9,10-epoxy-18-hydroxyoctadecanoic acid^a^	0.2 ± 0.1
25.7	Octadecane-1,9(10),18-triol	9,10-epoxy-18-hydroxyoctadecanoic acid	43.7 ± 0.4
31.1	Octadecane-1,9,10,18-tetraol	9,10,18-trihydroxyoctadecanoic acid	31.3 ± 0.5
31.7	Octadecanetetraol isomer ^b^	Trihydroxyoctadecanoic acid ^b^	3.0 ± 0.1
33.1	Unidentified monomer	-	0.9 ± 0

Values are expressed as the mean value ± SD (*n* = 3). ^a^ Position isomers of 9,10-epoxy-18-hydroxyoctadecanoic acid with differences in the position of hydroxylation (not determined). ^b^ Isomers of 9,10,18-trihydroxyoctadecanoic acid with differences in the position of hydroxylation (not determined).

TW was the second major constituent of the CM next to cutin, comprising 34.7% (w/w) of the CM (Table 1). The extraction of TW did not significantly change the CM thickness (Figure 1c); however, it drastically decreased the density of CM from 615 mg cm^-3^ to 434 mg cm^-3^ and produced a smooth surface morphology (Figure 1d,e). The predominant component in TW was ursolic acid (62.1%) (Figure 2b), a type of pentacyclic triterpenoid, followed by oleanolic acid (19.8%), which is a position isomer of ursolic acid (Table 3). Subsequently, the non-polar wax fraction (HW) was obtained via the *n*-hexane extraction of CM. The HW comprised 11.0% of the CM. From the GC/MS analysis, the HW was found to be predominantly composed of higher aliphatic compounds of alcohols and alkanes with chain lengths of C24 - C30 (Table 3).

**Table 3:** Composition of wax isolated from *Fuyu* persimmon fruits.

*Rt* (min)	Composition	Total wax (TW, %)	Hexane-soluble wax (HW, %)
	*Fatty acids*		
7.4	Tetradecanoic acid	0.3 ± 0.1	2.4 ± 0.3
11.8	Hexadecenoic acid	0.4 ± 0.1	11.4 ± 2.2
12.4	Hexadecanoic acid	0.9 ± 0	6.9 ± 1.4
17.1	Octadecadienoic acid	0.7 ± 0.2	6.7 ± 0.9
17.3	Octadecenoic acid	1.2 ± 0.2	9.3 ± 2.4
37.8	Hexacosanoic acid	2.7 ± 0.2	0
41.8	Octacosanoic acid	0.5 ± 0.1	0
	*Alkanes*		
16.6	Docosane	0	0.5 ± 0.4
19.3	Tricosane	0	1.1 ± 1.0
24.7	Pentacosane	0.1 ± 0	2.1 ± 1.3
27.3	Hexacosane	0	0.3 ± 0.2
29.8	Heptacosane	0.2 ± 0	1.4 ± 0.2
32.2	Octacosane	trace	0.3 ± 0.1
34.5	Nonacosane	0.1 ± 0	2.8 ± 0.3
39.0	Hentriacontane	0	0.5 ± 0.3
42.0	Dotriacontane	0	0.2 ± 0.3
43.7	Tritriacontane	0	0.5 ± 0.3
	*Alkanols*		
35.7	Hexacosanol	2.5 ± 0.3	3.3 ± 2.2
40.0	Octacosanol	2.8 ± 0.1	3.6 ± 0.9
44.1	Triacontanol	1.5 ± 0.1	1.7 ± 0.4
	*Triterpenoids*		
44.3	Beta sitosterol	1.1 ± 0.1	7.4 ± 1.9
48.7	Oleanolic acid	18.3 ± 0.7	10.0 ± 2.7
49.5/49.7	Ursolic acid	57.5 ± 3.7	8.7 ± 2.1
	Total unidentified	9.2 ± 3.7	25.9 ± 2.9

Values are expressed as the mean value ± SD (*n* = 3).

The polysaccharide and cutan contents were as low as 2.7% (w/w) and 2.0% (w/w), respectively. The predominant monosaccharide was glucose (55.5%, w/w), followed by relatively high levels of hemicellulosic and pectic sugars, such as arabinose (14.5%, w/w) and mannose (11.8%, w/w) (Table 4). These polysaccharides may be a component of the cell walls that incorporated into the cutin matrix during the deposition of cutin onto the exterior of the epidermal cells.

**Table 4:** Monosaccharide composition of CM isolated from *Fuyu* persimmon fruits.

Monosaccharide	Relative composition (wt %)
Arabinose	14.5 ± 0.5
Rhamnose	4.0 ± 0.3
Galactose	7.9 ± 1.8
Glucose	55.5 ± 0.4
Xylose	6.5 ± 1.0
Mannose	11.8 ± 0.4

Values are expressed as the mean value ± SD (*n* = 4).

### CP/MAS ^13^C-NMR analysis

The chemical distribution of the CM was further analyzed using CP/MAS ^13^C-NMR, and typical spectra of the CM, DCM and RW of *Fuyu* are shown in Figure 2c. The cutin signals that appeared in the NMR spectrum of DCM were assigned according to previously published spectral data for cutin isolated from limes and berries, as listed in Table 5 [24-26]. The assignments of the ursolic acid carbon signals that appeared in the RW spectrum are listed in Table 6, which are based on previous spectral data of ursolic acid [27] and higher aliphatic compounds [28] obtained using liquid state ^13^C-NMR. The spectrum for DCM predominantly contained carbon signals due to the alkyl chains and carbonyl carbons of cutin and that for RW consisted of signals due to a mixture of triterpenoids and higher aliphatic compounds. The spectrum for CM consisted of superimposed DCM and RW spectra. The NMR spectroscopic results were strongly consistent with the results from the chemical composition analysis of *Fuyu* CM described above.

**Table 5:** Assignments of carbons related to cutin in the solid state CP/MAS ^13^C-NMR spectra of CM and DCM of *Fuyu* persimmon fruits and their proton spin lattice relaxation times in the rotating and laboratory frames.

		*T*_1ρ_^H^ (ms)		*T*_1_^H^ (s)	
Assignment ^a^	Chemical shift (ppm)	Cutin in CM (fast/slow)	Cutin in DCM (fast/slow)	Cutin in CM	Cutin in DCM
(*C*H_2_)_*n*_	30	3.2/7.1	2.2/4.8	0.80	0.60
*C*HOCH	56	4.3/10.1	2.9/6.1	0.80	0.49
*C*H_2_OCOR, *C*H_2_OH	64	2.3/6.9	2.7/6.9	0.72	0.48
*C*HOH	72	3.9/9.1	3.1/8.0	0.65	0.53
*C*HOH, *C*HOCOR	74	2.8/9.2	2.9/8.1	0.60	0.52
C=O	173	3.4/13.0	2.9/8.1	0.48	0.41

^a^ Corresponding monomeric structures are shown in Figure 2a.

The results are expressed as the mean value of duplicate analyses.

**Table 6:** Assignments of carbons related to wax in the solid state CP/MAS ^13^C-NMR spectra of CM and RW of *Fuyu* persimmon fruits and their proton spin lattice relaxation times in the rotating and laboratory frames.

		*T*_1ρ_^H^ (ms)		*T*_1_^H^ (s)	
Assignment ^a,b^	Chemical shift (ppm)	Wax in CM (fast/slow)	Wax in RW	Wax in CM	Wax in RW
24, 25, 26, 29	15-18	4.5/9.9	12	0.88	1.2
6	19	4.5/11	13	0.61	1.2
*C*H_2_^c^ & 7	33	3.8/7.7	8.1	1.1	1.7
10, 22	38	3.3/10	15	0.76	1.2
1, 4, 8, 19, 20	40	7.0/11	16	0.89	1.2
14	42	5.0/13	14	0.72	1.2
9, 17	48	5.6/12	16	0.74	1.2
5, 18	53-56	4.3/11	17	0.80	1.2
3	79	3.2/10	14	0.62	1.3
12	126	2.6/8.7	15	0.77	1.2
13	139	2.6/11	11	0.81	1.4
28	186	2.3/9.8	10	1.1	1.3

^a^ Corresponding monomeric structures are shown in Figure 2b. ^b^ Relaxation times of carbons of C-2, 11, 15, 16, 21, 23, 27, 30 of wax are not presented due to their overlap with the cutin signals. ^c^ The signal at 33 ppm originated mainly from the CH_2_ chain of higher aliphatic compounds with a slight contribution from the C-7 of ursolic acid.

The results are expressed as the mean value of duplicate analyses.

### Measurement of proton spin-lattice relaxation time in the rotating frame (*T*_1ρ_^H^) of *Fuyu* CM

Based on the signal assignments, the molecular mobility of carbons in the cutin and wax components at time scales of *T*_1ρ_^H^ (corresponding to ≤ 5 nm scale of molecular mobility) was then measured. The *T*_1ρ_^H^ value for cutin was measured at 30 ppm. In the case of wax, signals at 17 ppm and 33 ppm were used as representative signals of triterpenoids and higher aliphatic compounds, respectively. The *T*_1ρ_^H^ values for fast and slow relaxation times were determined for carbons of cutin (in CM and DCM) and wax (in CM) (Tables 5 and 6) due to their biphasic relaxation decay (Figure 2d).

Cutin, triterpenoids and higher aliphatic compounds presented clearly different relaxation patterns when they were separated as DCM and RW. The *T*_1ρ_^H^ for cutin in DCM had a faster decay due to its high molecular mobility, whereas the signals of triterpenoids in RW attenuated slowly because of their higher crystallinity. However, because of the formation of a wax-cutin composite in the CM, the relaxation behaviors of the triterpenoids became faster and that of cutin became slower at the same time. This weak convergence suggested the presence of a small cross-interaction between these components at the time scale for *T*_1ρ_^H^. In contrast to triterpenoids, the carbons of higher aliphatic compounds did not show any clear interaction with cutin under the time scale for *T*_1ρ_^H^.

Additionally, the biphasic relaxation decay of cutin indicated the existence of at least two domains with different mobilities in the cutin network. Similar to cutin, the carbons of the triterpenoids in CM exhibited biphasic relaxation decay when they were incorporated in cutin (Table 6 and Figure 2d).

### Measurement of proton spin-lattice relaxation time in the laboratory frame (*T*_1_^H^) of *Fuyu* CM

The values of *T*_1_^H^ were subsequently measured (Tables 5 and 6). At this time scale, all the carbons in cutin and wax relaxed linearly. Similar to the *T*_1ρ_^H^ analysis, the cutin in DCM exhibited a fast decay, whereas the triterpenoids and higher aliphatic compounds in RW exhibited a delayed decay (Figure 2e). When these components formed a composite in CM, however, the relaxation behaviors of the triterpenoids and cutin completely coincided with each other, revealing an apparent spin diffusion process between these components at relaxation times of 0.7 to 1.0 s. Identical behaviors were also observed from the different carbons assignable to triterpenoids (Table 6). In contrast, the higher aliphatic compounds only showed a weak interaction at this scale, confirming their lower compatibility with cutin (Figure 2e).

The effective domain sizes (*L*) of cutin and the triterpenoids were then estimated from the values of *T*_1ρ_^H^ and *T*_1_^H^. The results indicated that the compatibility of cutin and the triterpenoids was insufficient at the *T*_1ρ_^H^ scale ≤ 2-5 nm; however, these components were compatible at the *T*_1_^H^ scale ≤ 20-24 nm, as observed in the completely consistent relaxation pattern obtained during the *T*_1_^H^ analysis.

### Wide-angle X-ray diffraction analysis of *Fuyu* CM

**Figure 3:**
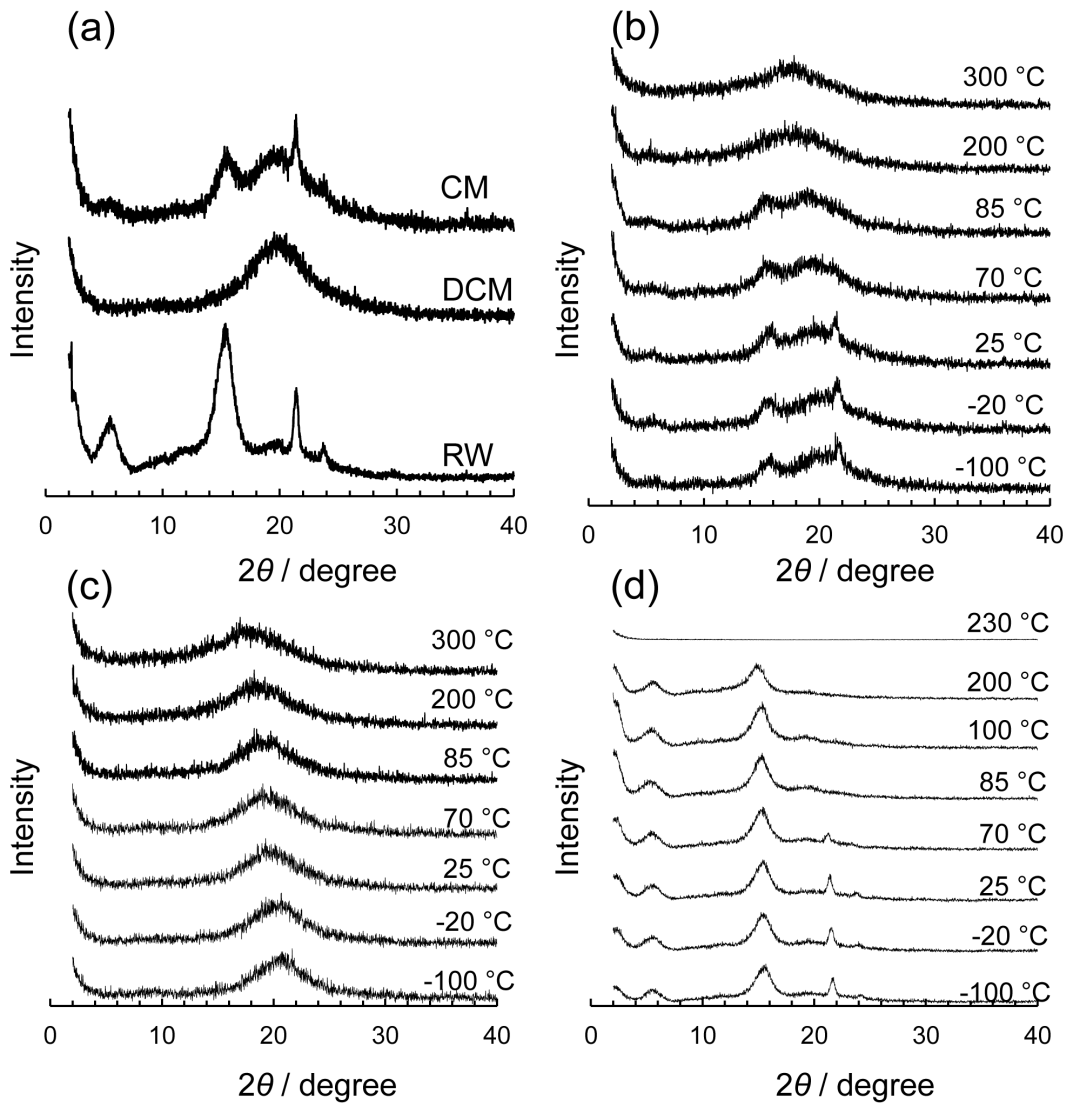
Wide angle X-ray diffraction (WAXD) patterns of CM, DCM and RW. (a) CM, DCM and RW at 25°C, and (b) CM at -100 °C - 300 °C. (c) DCM at -100 °C - 300 °C and (d) RW at -100 °C - 230 °C.

Diffraction profiles of the crystalline components in the CM, DCM and RW were obtained using WAXD (Figure 3a). The isolated DCM exhibited an amorphous halo at approximately 20.0 °, which confirmed the completion of wax extraction. Furthermore, the DCM did not present a cellulose diffraction peak, coinciding with its low content in the CM. RW showed clear diffraction peaks at 6.1 °, 15.9 °, 22.0 ° and 24.3 °. According to Casado et al. [29] and based on the authentic standard of ursolic acid, the former two broad diffraction peaks at 6.1 ° and 15.9 ° correspond to triterpenoids and the latter sharp diffraction peaks at 22.0 ° and 24.3 ° correspond to higher aliphatic compounds. The sizes of the crystalline components in wax (*D*) were then obtained using Scherrer’s equation, and the minimum sizes of each component were estimated to be 3.7 to 5.3 nm for triterpenoids and 9.6 to 13.6 nm for higher aliphatic compounds. The wide half width of the peaks of the triterpenoids indicated their low degree of molecular orientation. In contrast, the sharp peaks of the higher aliphatic compounds indicated that they are in a highly ordered crystalline state.

Subsequently, the temperature dependency of the crystallinity of CM, DCM and RW was investigated at temperatures between -100 °C and 300 °C (Figure 3 b-d). The amorphous halo of cutin observed in DCM shifted to a lower diffraction angle with increasing temperature due to thermal expansion (Figure 3c). The diffraction peaks of the higher aliphatic compounds in CM and RW disappeared above 70 °C, which corresponds to their melting point [29], whereas those of the triterpenoids disappeared at temperatures greater than their melting points (283-285 °C) at approximately 300 °C (Figure 3 b and d).

### Tensile test of Fuyu CM

**Figure 4:**
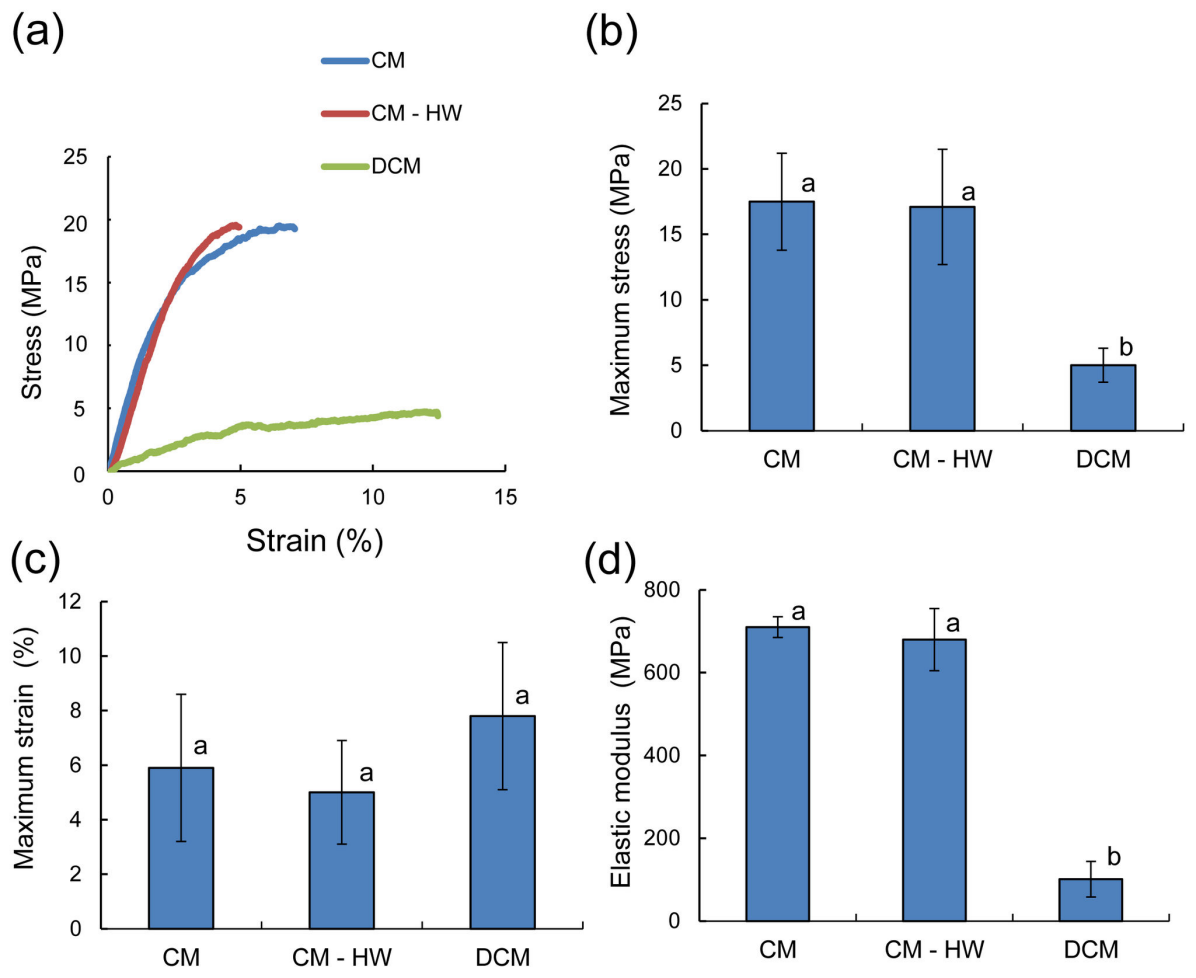
Tensile properties of cuticular membranes of *Fuyu* persimmon fruits. (a) Typical stress-strain curves of the CM, CM – HW and DCM and the corresponding values of (b) maximum stress, (c) maximum stress and (d) elastic modulus. Different letters indicate a significant difference at *P* < 0.01 (*n* = 8-9) based on Tukey’s test.

Because of the importance of the wax components in the CMs of *Fuyu* fruits, the mechanical properties of CM and DCM were further compared using tensile tests, which is similar to the system that Wiedemann et al. [15] and Bargel et al. [10,30] previously used. Typical stress-strain (S-S) curves are shown in Figure 4a, and the corresponding maximum stress, maximum strain and elastic modulus are shown in Figure 4b-d. The S-S curve for CM exhibited a steep and linear elastic deformation followed by plastic deformation. The removal of only HW did not change the mechanical properties of the membrane; however, thorough dewaxing using a solution of chloroform and methanol significantly eliminated the elastic phase, which was accompanied by an increase in plastic deformation. These results clearly indicate the significant importance of wax, especially triterpenoids, for providing the mechanical toughness to *Fuyu* CMs.

### Dynamic mechanical analysis of *Fuyu* CM

**Figure 5:**
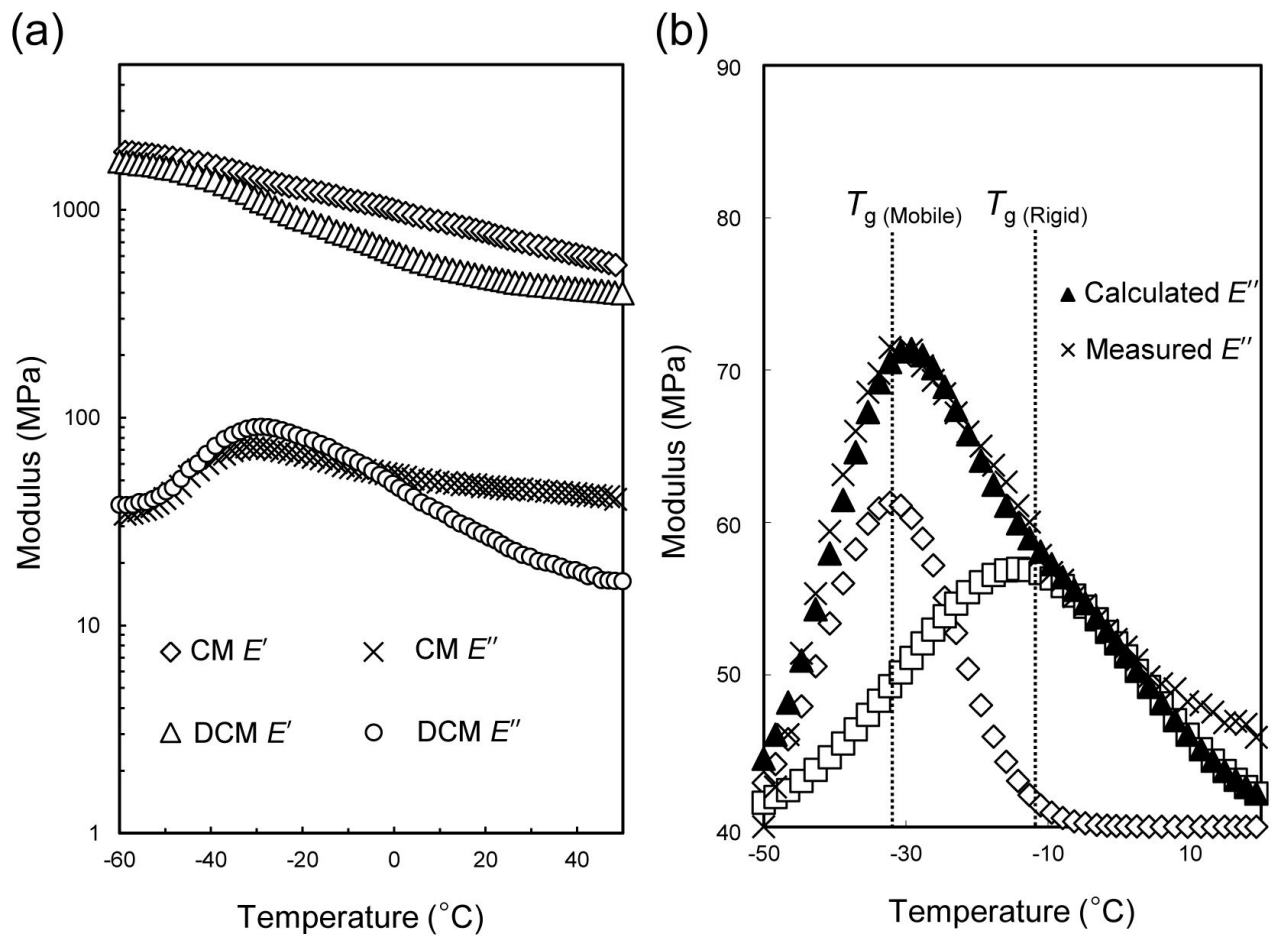
Dynamic mechanical analysis of CM and DCM isolated from *Fuyu* persimmon fruits. (a) DMA plots for the storage elastic modulus (*E*′) and loss elastic modulus (*E*′′). (b) *E*′′ of DCM analyzed by wave-form separation using a Gaussian function.

The temperature dependency of the mechanical properties was analyzed using DMA at temperatures ranging from -60 °C to 50 °C (Figure 5a). The storage elastic modulus (*E*′) represents the elasticity of the CM. The *E*′ values of CM showed no significant changes; however, that of DCM decreased at -42 °C. The loss elastic modulus (*E*″) represents the viscoelasticity of the material. The CM showed an increased *E*″ at temperatures above -33 °C, while that for DCM gradually decreased at temperatures above -30 °C, indicating that the presence of wax also affects the viscoelasticity.

The *T*_g_ for cutin was determined from the *E*″ peak of DCM. Due to the unequal distribution of the peak shape, the *E*″ curve could be divided into two peaks by waveform separation using a Gaussian function to obtain the glass transition temperatures (*T*_g_) for the mobile and rigid domains centered at -32 °C and -14 °C, respectively (Figure 5b). The *T*_g_ range was consistent with that for tomato CM determined using DSC [31]. The two *T*_g_s supported the existence of two domains with different molecular mobilities in the cutin network.

## Discussion

### CMs of *Fuyu* persimmon fruit are abundant in wax

The composition of the fruit CM of *Fuyu* was considerably different from that of tomato, which has frequently been used as a model plant for analyzing the biomechanical properties of CMs [8]. The *Fuyu* fruit CMs were abundant in wax and cutin, with a very low amount of polysaccharide and cutan. Therefore, the wax and cutin network appeared to be important for the mechanical strength of the *Fuyu* fruit cutin matrix. The wax was primarily composed of ursolic and oleanolic acids, with smaller amounts of higher aliphatic compounds. Both acids are types of pentacyclic triterpenoids and are widely distributed in the plant kingdom [4] as well as in the leaves and fruit flesh of persimmons [32,33]. In many plant species, the principal components in wax are free alcohols, β-diketones and esters; however, triterpenoids are occasionally major constituents of wax from fruits such as apples, grapes, and leaves of eucalyptus [4,21,22,29,34]. The complete extraction of wax from the CMs decreased their densities by 30%; therefore, waxes significantly contribute to fill the cutin network to provide a denser membrane. Buschhaus et al. recently reported the localization of wax components in the CM of *Ligustrum* *vulgare* leaves, in which the epicuticular wax entirely consisted of higher aliphatic compounds while the intracuticular was consisted of triterpenoids predominant in ursolic and oleanolic acids [18]. A similar trend has also been reported for CMs abundant in triterpenoids [35].

The cutin network was predominantly constructed by C18 cutin monomers (Figure 2a). Epoxidized and hydroxylated C18 monomers are ubiquitously found in, e.g., tea leaves ( *Camellia* *sinensis* ) [23] and a type of mangrove ( *Sonneratia* *alba* ) [6]; however, the composition of the cutin was considerably different from that of tomato and lime, which were predominant in C16 monomers and often used as model CMs [36]. The dominance of wax and cutin in the CM of *Fuyu* fruit with its very low polysaccharide and cutan content provides an advantage for analyzing the function of wax and cutin in CMs in detail while discriminating the effects from other components.

### Triterpenoids in wax and cutin construct a nanocomposite structure in the *Fuyu* CM

In this study, we employed the NMR technique to analyze the compatibility of wax components within the cutin network, and this technique is usually used in the analysis of synthetic polymers [37] with the assistance of WAXD. The NMR spectrum of the *Fuyu* fruit CM was quite different from those from tomato and lime fruit CMs. However, the NMR spectrum of the *Fuyu* fruit CM was rather similar to the spectrum of apple fruit CM [38], reflecting the abundance of triterpenoids in the wax (Figure 2c). The triterpenoids in CM showed clear spin diffusion with cutin, as shown by the *T*_1_^H^ analysis, demonstrating the high compatibility of cutin with triterpenoids. The compatibility of cutin and triterpenoids was further estimated to be in the range of less than 20 to 24 nm. The board WAXD pattern indicated that the triterpenoids have low crystallinity, and the minimum crystalline size was estimated to be 3.7 to 5.3 nm. The *T*_1ρ_^H^ value and WAXD analysis revealed that these triterpenoids were dispersed in the CM with low molecular ordering. The crystalline and amorphous structures of *Fuyu* wax was consistent with that previously proposed for grape wax by Casado et al. [29]. Based on the above results, the cutin and triterpenoid in wax was concluded to construct a nanocomposite in the CMs of *Fuyu* fruits.

In contrast, the relaxation behavior of the higher aliphatic compounds showed no difference between RW and CM at the relaxation time scale for *T*_1ρ_^H^. Their relaxation behaviors came slightly closer at the mixing scale observable by *T*_1_^H^ analysis, showing the presence of a small degree of compatibility at a scale below 40 nm. The results indicated that the cutin network was less compatible with higher aliphatic compounds than with triterpenoids. This result is most likely because the higher aliphatic compounds are primarily located at the outer region of the cutin network, whereas the triterpenoids are embedded in the cutin matrix and have close interactions with cutin [18,35].

NMR techniques have previously been used to investigate the nanomechanical properties of tomato and lime CMs to evaluate the effects of hydration and temperature [28,39,40] as well as the molecular structures of cutin oligomers [25,41,42]. In the case of *Citrus* CM, for instance, Garbow et al. (1990) reported that cutin and higher aliphatic compounds in key lime ( *Citrus* *aurantifolia* ) fruit wax have spin diffusions at mixing scales detectable by *T*_1ρ_^H^ measurements [28]. In contrast, Reynhardt and Riederer reported that wax molecules in bitter orange ( *Citrus* *aurantium* ) leaves do not influence the molecular dynamics of the matrix membrane [43]. Differences in the molecular mobilities of cutin and wax between persimmon and *Citrus* may be attributed to differences in the chemical compositions of both cutin and wax. *Citrus* cutin is predominantly composed of hydroxylated and oxygenated C16 monomers, and the wax content is as low as 4% [43] and predominantly composed of higher aliphatic compounds with chain lengths of C25 to C53. These differences may indicate that wide variations in the chemical constituents and molecular arrangements of CM components occur depending on the plant species.

### Heterogeneous nanomechanical properties of cutin

The *T*_1ρ_^H^ and DMA analyses of DCM further indicated the heterogeneous nanomechanical properties of the cutin network. The biphasic relaxation pattern of cutin during the *T*_1ρ_^H^ analyses of DCM and CM indicated the presence of at least two domains with different mobilities in the cutin network at a few-nanometer scale (Figure 2d). These domains were presumed to have branch structures of the cutin network with motional constraints at particular esterification in-chain hydroxyl groups [24]. Two overlapping *T*_g_ peaks in the *E*′′ of the DMA plot are also consistent with the presence of two domains below ca. 15 nm (Figure 5b). Similar to *Fuyu* CM, the presence of multiple *T*_g_ values was also found in tomato [31]. Deshmukh et al. reported that most of the mid-chain hydroxyls in tomato CM appeared to be unesterified in addition to ester-linked mid-chain hydroxyls [25]. Graça et al. recently reported that C16 monomers in tomato fruit CM were mainly polymerized in their in-chain hydroxyl groups and constructed a reticulate structure [44]. In contrast, C18 monomers in *H*. *helix* were linear head-to-tail structures and constructed a lamellar layer. In the case of *Fuyu* CM, a junction in the in-chain branch structures provides a rigid domain with low molecular mobility at scales below ca. 15 nm. However, the *T*_1_^H^ analysis exhibited a linear decay in the signal intensity of cutin carbon, indicating that these structural restraints of in-chain branching might be averaged at a scale below ca. 40 nm, which is the scale measurable by *T*_1_^H^ analysis.

The combination of NMR, WAXD and DMA analyses provided an integrated understanding of the nanostructure of *Fuyu* persimmon fruit CM. Despite recent progress in the biosynthesis of cutin and wax from a genetic perspective [3,45], structural information on cutin and composite structures with other components at the microscopic scale were lacking. The present polymer analyses may also be applicable for investigating dynamic nanostructure formation along with cutin and wax deposition.

### Importance of triterpenoid as a filler for the mechanical properties of *Fuyu* CM

The functions of wax on the mechanical properties of CM were investigated using a tensile test. The S-S curve of *Fuyu* CM was clearly divided into two zones: elastic and plastic deformation (Figure 4a). The shape of the curve was very similar to that of *Nerium* *oleander* ; however, it was considerably different from that of tomato and *Yucca* *aloifolia* , which only showed a linear elastic phase [15]. The removal of triterpenoids rather than the higher aliphatic compounds from the CM significantly reduced its density, elasticity, and strength while increasing its viscoelasticity, indicating that the triterpenoids in wax have filler effects on the mechanical properties of *Fuyu* CMs. Waxes predominantly composed of higher aliphatic compounds are known for their filler effects by reducing the free volume and segmental mobility of the cutin network in tomato CMs, similar to flavonoids [13]. Therefore, the removal of wax increased the flexibility of the CMs of tomato fruit [11] and mangrove leaf [6]. In the present case, the triterpenoids showed higher compatibility with cutin rather than the higher aliphatic compounds at the 20-24 nm scale, which provided the membrane with significantly improved elasticity.

DMA also supported these filler effects based on both the storage elastic modulus and the loss elastic modulus of the CM (Figure 5a). By removing TW, DCM showed clear weakening in both moduli, specifically at temperatures greater than the *T*_g_ of cutin. Therefore, depositions of wax in *Fuyu* CM were confirmed to be important for facilitating greater defensive properties against various biotic and abiotic mechanical stresses.

## Conclusions

This study demonstrated the existence of interactions between cutin and triterpenoids in cuticular wax as well as their impact on the mechanical properties of CM isolated from wax-rich fruits of *Fuyu* persimmon. The CM was rich in cutin (48.4%), which was primarily composed of C18 hydroxylated and epoxy-hydroxylated fatty acids. The CM contained significantly large amounts of wax (34.7%), mainly composed of triterpenoids, such as ursolic and oleanolic acid. The tensile tests revealed that wax is significantly important for maintaining the mechanical properties of the CM. Cutin and triterpenoids were estimated to be compatible at scales below 20 to 24 nm. The WAXD analysis indicated that the triterpenoids construct a low-ordered crystal in the size range of at least 3.7 to 5.3 nm, and these crystals may fill the gaps as intracuticular wax in the cutin matrix. The existence of molecular restriction in the cutin matrix was also observed at scales below ca. 15 nm from the *T*_1ρ_^H^ measurement and DMA. In summary, the triterpenoids in wax were important as nanofillers for strengthening the CM.
